# Reduction of Acid-Fast and Non-Acid-Fast Bacteria by Point of Use Coagulation-Flocculation-Disinfection

**DOI:** 10.3390/ijerph121114420

**Published:** 2015-11-13

**Authors:** Lisa M. Casanova, Mark D. Sobsey

**Affiliations:** 1 Division of Environmental Health, School of Public Health, Georgia State University, P.O. Box 3995 Atlanta, GA 30302, USA; 2 Department of Environmental Sciences and Engineering, Gillings School of Global Public Health, University of North Carolina Chapel Hill, Rosenau Hall, Room 148, 135 Dauer Drive, Campus Box 7431, Chapel Hill, NC 27599, USA; E-Mail: mark_sobsey@unc.edu

**Keywords:** coagulation, flocculation, disinfection, *Mycobacterium*, point-of-use, water treatment

## Abstract

Point of use (POU) household water treatment is increasingly being adopted as a solution for access to safe water. Non-tuberculous Mycobacteria (NTM) are found in water, but there is little research on whether NTM survive POU treatment. Mycobacteria may be removed by multi-barrier treatment systems that combine processes such as coagulation, settling and disinfection. This work evaluated removal of a non-tuberculous *Mycobacterium* (*Mycobaterium terrae*) and a Gram-negative non-acid-fast environmental bacterium (*Aeromonas hydrophila*) by combined coagulation-flocculation disinfection POU treatment. *Aeromonas hydrophila* showed 7.7 log_10_ reduction in demand free buffer, 6.8 log_10_ in natural surface water, and 4 log_10_ reduction in fecally contaminated surface water. Turbidity after treatment was <1 NTU. There was almost no reduction in levels of viable *M. terrae* by coagulant-flocculant-disinfectant in natural water after 30 minutes. The lack of Mycobacteria reduction was similar for both combined coagulant-flocculant-disinfectant and hypochlorite alone. A POU coagulant-flocculant-disinfectant treatment effectively reduced *A. hydrophila* from natural surface waters but not Mycobacteria. These results reinforce previous findings that POU coagulation-flocculation-disinfection is effective against gram-negative enteric bacteria. POU treatment and safe storage interventions may need to take into account risks from viable NTM in treated stored water and consider alternative treatment processes to achieve NTM reductions.

## 1. Introduction

Household water treatment is increasingly being adopted as a solution for households and communities without access to safe water sources. Households with access to unsafe sources frequently also carry water from the source and store it in the home. While a number of point-of-use (POU) household water treatment technologies have demonstrated effectiveness against microbial pathogens, storage remains a critical link in the provision of safe water access at the household level [1,2]. Renewed multiplication of bacteria that either survive the treatment process or are introduced into water can result in recontamination that may pose health risks [3]. The formation of biofilms on the interior of storage containers may provide an environment for potentially pathogenic bacteria to grow and recontaminate treated water as they are released from the biofilm [4]. 

Members of the genus *Mycobacterium* other than *M. tuberculosis*, collectively known as non-tuberculous Mycobacteria (NTM), are recognized causes of illness in immunocompromised people, including people living with HIV/AIDS [5,6]. These bacteria are acid-fast, with cell walls containing mycolic acids, which non-acid fast bacteria lack. This includes the *Mycobacterium avium* complex (MAC). Treated water has been implicated as a possible source of MAC illness in individuals with HIV/AIDS and others [5]; *M. avium* is on the US Environmental Protection Agency’s Contaminant Candidate List of microbial water contaminants under consideration for regulation because of their health effects [7]. 

In developing countries, high HIV prevalence often coexists with lack of access to safe water [8,9]. In these countries, the potential risk of MAC from water is largely unexplored. In countries with near-universal infrastructure coverage, the NTM are found in treated water and distribution systems [10,11], but there is little research on whether NTM survive POU water treatment processes, potentially surviving to contaminate stored water. If NTM are present in untreated water and survive the treatment process, they may grow in biofilms in stored water. These biofilms could represent a possible pathway of exposure for household members. 

Mycobacteria may be removed by multi-barrier water treatment systems that combine conventional water treatment processes such as coagulation, settling and disinfection, including those applied at POU. POU treatments are often evaluated for efficacy in the laboratory using representative gram-negative, non-acid-fast enteric bacteria such as *E. coli* and *Raoultella terrigena*, but it is possible that NTM survive treatment processes that are effective against such test bacteria. One such multi-barrier treatment is composed of a ferric coagulant, an alkaline agent, flocculation aids, and a chlorine-based disinfectant. To our knowledge, POU treatments such as combined coagulation-flocculation-disinfection have not been evaluated for efficacy against NTM. Therefore, the purpose of this work is to determine whether (1) a representative non-tuberculous *Mycobacterium* (*M. terrae*) and (2) a representative gram-negative non-acid-fast environmental bacterium (*A. hydrophila*) can be reduced by a combined coagulation-flocculation disinfection POU treatment. *A. hydrophila* was the gram-negative potentially enteric bacterium selected for this study because it can proliferate in water and possibly regrow in stored water.

## 2. Experimental Section

Pure cultures of *A. hydrophila* (ATCC # 7966) were grown in typtic soy broth at 37 °C on a shaker flask for 24 hours. Cultures were centrifuged twice at 16,000 rpm for 10 minutes, and the pellet was washed and resuspended in sterile 0.9% NaCl for inoculation into test waters. *Mycobacterium* experiments used pure cultures of *M. terrae* (kindly provided by University of North Carolina Hospitals). Cultures were prepared by inoculating 100 mL of Middlebrook 7H9 broth with 100 μL of *M. terrae* culture maintained in frozen stocks. Cultures were incubated at 37 °C on a shaker flask for 5 days until the OD520 of the culture reached 0.3–0.4. The culture was centrifuged twice (3000 × *g,* 10 °C, 30 minutes), and the pellet washed and resuspended in 20 mL sterile 0.9% NaCl. 

Oxidant demand free (ODF) buffer (pH 7) was prepared as previously described [12]. Natural surface water was obtained from Botany Pond, a natural lake in Chapel Hill, NC (USA), and used within 24 hours of collection. For a “worst case” test water, fecally contaminated surface waters were created by spiking Botany Pond water with 10% or 20% (v/v) primary wastewater effluent obtained from Orange Water and Sewer Authority wastewater treatment facility, Chapel Hill, NC. Experiments with *A. hydrophila* were conducted in ODF buffer, natural surface water, and fecally contaminated surface water. Experiments with *M. terrae* were conducted in natural surface water. 

The coagulant-flocculant-disinfectant came as a pre-packaged product intended to treat 10 L of water. All experiments were carried out at 25 °C. A 10 L volume of test water was spiked with 10 mL of test bacteria culture, and the water was stirred for 30 s to distribute the organisms. A 15 mL sample was taken to determine the initial concentration of microorganisms (time 0). The coagulant-flocculant disinfectant was added according to the manufacturer’s instructions. Coagulant-flocculant-disinfectant was added, the water was stirred vigorously for 30 s, and slow stirring was continued for 5 min. The water was then allowed to sit for an additional 25 minutes to allow flocculation, settling, and disinfectant contact time. Hypochlorite was prepared fresh each time as stock solution in ODF buffer, added to water at a final concentration of 5 mg/L, and allowed 30 minutes contact time. 

Samples for microbiological analysis were taken from the middle of the water column at 30 s, 5, 10, 20, and 30 min, and immediately neutralized with 0.1% sodium thiosulfate. Samples for residual chlorine analysis were taken at the same time and residual chlorine was measured immediately using N,N-diethyl-p-phenylenediamine titration [13]. Turbidity was measured using a Hach turbidimeter (Hach Co, Loveland, CO, USA). 

*A. hydrophila* was enumerated according to EPA Method 1605 [14]. *M. terrae* samples were centrifuged (3000 × *g*, 10 °C, 30 min), and the pellets resuspended in 2 mL 0.9% NaCl. To reduce overgrowth of slow growing Mycobacteria by rapidly growing contaminating organisms, samples were decontaminated using the N-acetyl-L-cysteine method [15]. Samples were diluted and spread plated on Middlebrook 7H11 agar containing 10 mg/L Amphotericin B (to suppress growth of fungi). Plates were incubated for 14 days at 35 °C in a 10% CO_2_ atmosphere. Counts were expressed as CFU/mL. Data analysis was done using GraphPad Prism 5 (GraphPad, LaJolla, CA, USA).

## 3. Results and Discussion

**Figure 1:**
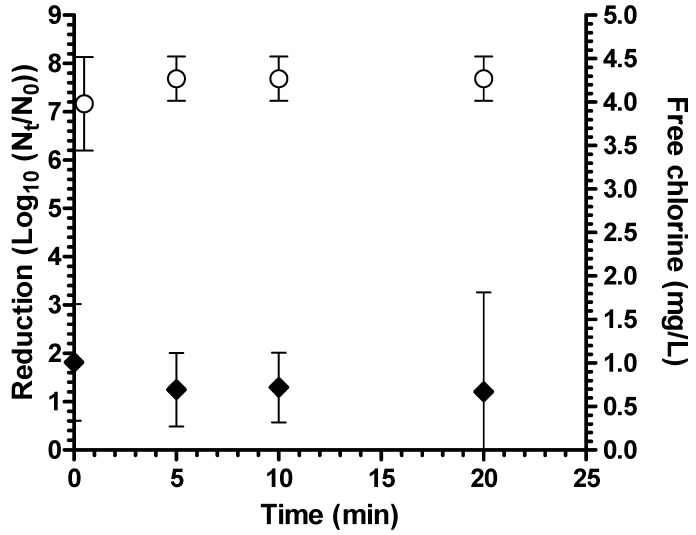
Reduction of *A. hydrophila* in ODF buffer (*n* = 4 experiments) (white circles = bacterial reduction; black diamonds = free chlorine residual; bars = 95% confidence interval).

**Figure 2:**
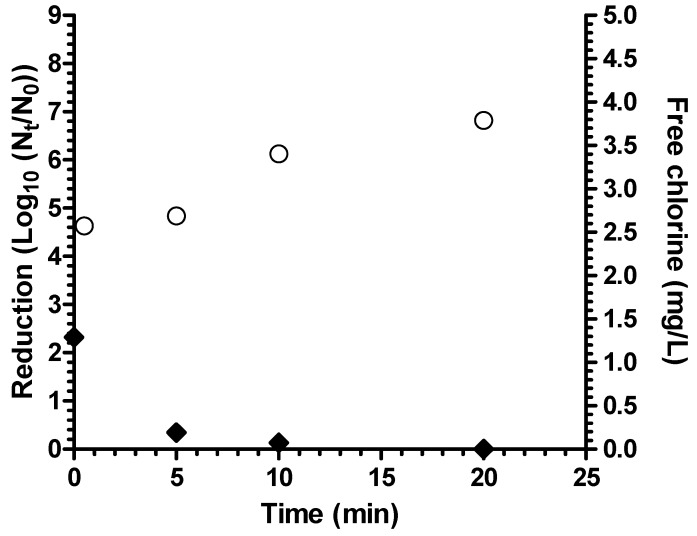
Reduction of *A. hydrophila* in natural surface water (B, *n* = 2 experiments) (white circles = bacterial reduction; black diamonds = free chlorine residual; bars = 95% confidence interval).

The coagulant-flocculant-disinfectant was tested for performance against *Aeromonas hydrophila* in both ODF buffer (pH 7.3) and natural surface water (mean turbidity 2.6 NTU, mean pH 7.6). There was a mean 7.7 log_10_ reduction in ODF after 20 minutes of contact time (Figure 1). In natural surface water, the average log_10_ reduction was extensive at 6.8 log_10_ but significantly lower than in ODF water (*p* = 0.02 by student’s t test) (Figure 2). The lower log_10_ reduction in natural water is possibly due to the higher chlorine demand of this water as free chlorine concentration was under the detection limit (0.1 mg/L) in natural water after 20 minutes but was an average of 0.67 mg/L after 20 minutes in ODF. Nevertheless, bacterial reduction in natural surface water still exceeded EPA guidelines of 6 log_10_ [16]. 

**Figure 3:**
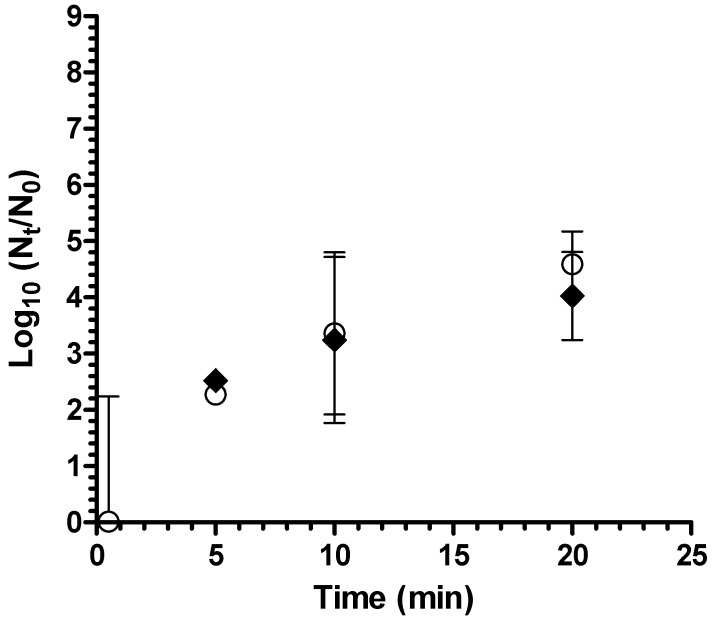
Reduction of *A. hydrophila* in fecally contaminated natural water (white circles = 10% primary effluent; black diamonds = 20% primary effluent; bars = 95% confidence interval).

Coagulant-flocculant-disinfectant achieved a 4 log_10_ reduction of *A. hydrophila* in test waters that simulated fecally contaminated surface waters as “worst case” water (average turbidity 8.0 NTU, average pH 7.4) (Figure 3), thereby meeting the 4 log_10_ highly protective bacteria reduction performance target of the World Health Organization [17]. There was no significant difference in log_10_ reduction between waters with 10% (v/v) and 20% primary effluent (*p* = 0.14). There was no detectable residual chlorine remaining after 20 minutes contact time in fecally contaminated worst case test water. For both natural and fecally contaminated worst case test waters, turbidity after treatment was <1 NTU. 

In contrast to the log_10_ reductions observed for *A. hydrophila*, there was almost no reduction in levels of viable *M. terrae* by coagulant-flocculant-disinfectant in natural water after a contact time of 30 minutes (Table 1). Because this treatment is a combination of coagulation-flocculation and disinfection, hypochlorite alone at a target concentration of 5 mg/L was also tested separately to confirm that the Mycobacteria were resistant to hypochlorite disinfection. The lack of Mycobacteria reduction was similar for both combined coagulant-flocculant-disinfectant and hypochlorite alone after 30 minutes contact time in natural water (*p* = 0.96).

**Table 1:** Reduction of *M. terrae* in natural water by coagulant-flocculant-disinfectant and free chlorine.

	log_10_ Reduction (95% CI)		Free Chlorine Residual (mg/L)	
	30 s	30 min	30 s	30 min
Coagulant-flocculant-disinfectant	0.02	0.23 (0.31–0.77)	5.87	4.46
Hypochlorite	−0.65	0.21 (−3.1–3.5)	0.96	0.57

A POU coagulant-flocculant-disinfectant treatment that effectively reduced *A. hydrophila* from natural surface waters by 4–7 log_10_ was not effective in reducing Mycobacteria. In both ODF buffer and natural surface water, coagulant-flocculant-disinfectant achieved ~7 log_10_ reduction of *A. hydrophila*, exceeding the POU water treatment bacterial reduction levels of both EPA guidelines (>6 log_10_) and WHO targets (4 log_10_ reduction) [16]. In natural waters contaminated with human fecal waste, reductions were lower but still reached 4 log_10_ to meet the WHO performance target. These results reinforce previous findings that point of use coagulation-flocculation-disinfection is an effective treatment method against gram-negative enteric bacteria, including *A. hydrophila* [18]. 

Although USEPA microbial reduction performance guidelines for point-of-use water treatment technologies are applied to products sold in the U.S. market, there are more recent World Health Organization microbial reduction performance targets now available for developing countries as well. The WHO performance targets focus on the specification of three levels of microbial reduction performance to evaluate point-of-use technologies in ways that are appropriate to a wide variety of situations. The three WHO categories of microbial reduction performance are “highly protective” “protective” and “minimally protective” at 4, 3 and 2 log_10_ bacteria reductions, respectively [17]. In natural surface water the coagulant-flocculant-disinfectant met the 4 log_10_ reduction target for the “highly protective” category of point-of-use drinking water treatment technology. It also met this “highly protective” reduction target in highly fecally contaminated surface water, and was effective in reducing turbidity in water to less than 1 NTU in such test water. 

There are no specific guidelines for levels of Mycobacteria in drinking water treated at the point of use or by centralized facilities or for their reductions by treatment processes or systems. In this study the combined coagulant-flocculant-disinfectant treatment was not effective for reduction of *M. terrae*, a representative NTM, in natural surface water, a “worst case” fecally contaminated water or a high quality buffered water. The POU treatment performance comparison of *A. hydrophila* and *M. terrae* in this study also suggests that gram-negative indicator bacteria may be poor indicators of POU treatment performance against Mycobacteria. The low reduction of *M. terrae* over 30 minutes contact time by hypochlorite in this study is consistent with previous findings that *Mycobacterium* are highly resistant to chlorine disinfection [19]. Ct values for 99.9% reduction ranging from 50–200 have been reported for *M. avium* grown in standard media; Ct values are even higher for strains grown in low-nutrient conditions [20]. However, the low reduction of *M. terrae* by combined coagulant-flocculant-disinfectant in this study suggests that the coagulation-flocculation process also achieves low removal of NTM. 

Although there is evidence that conventional combined treatment processes are effective against Mycobacteria [10,11,21], there is little evidence on the effectiveness of coagulation processes alone in removing NTM from water. One study of coagulation alone found that at doses optimized for three different test waters, alum coagulation-flocculation achieved on average 0.7, 0.6, and 2.2 log_10_ removal, depending on test water, lower than removal of *E. coli* tested in parallel [22]. These NTM reductions were greater than those observed in this present study. However, the coagulant doses in the Wong study were optimized for the test waters using concentrations from 10–30 mg/L, whereas this study used a single, standard dose of coagulant for 10 L of water regardless of water quality. The hydrophobicity of the Mycobacterial cell surface suggests that Mycobacteria in water will attach to surfaces such as other particles, and thus processes that are effective for removing particles would be expected to also remove these microorganisms [23]. However, if the cells are not associated with particles, but instead exist as hydrophobic aggregates of bacteria in water, the charge destabilizing effects of a conventional coagulant may not work to cause these aggregates to coagulate and flocculate, leaving them in the water column after the coagulation, flocculation and settling processes. These bacteria aggregates may also contribute to disinfection resistance as has been previously observed for other bacteria [24]. It is possible that optimized coagulant doses might achieve higher reductions of NTM in water. However the extent to which the reductions would be sufficient to meet either EPA standards or WHO recommendations for the three different categories of protection, “highly protective”, “protective”, and “minimally protective”, levels of POU treatment performance, are now unknown and would require further study. 

NTM have been isolated from environmental waters in countries where people may use untreated surface water for drinking water supply [25]. Waterborne Mycobacteria typically compete poorly for nutrients in the presence of other organisms [23]. However, disinfection can disinfect competing organisms, selecting for genera that are more resistant to disinfection [10] and allowing Mycobacteria to grow preferentially in treated drinking water [23]. POU treatment with combined coagulation-flocculation-disinfection is highly effective against non-acid fast bacteria, including gram-negative environmental bacteria that are representative of many waterborne pathogens [18]. Ingestion is a potential route of exposure to NTM infection, particularly for people living with HIV [5,6], who are also at highest risk for diarrheal disease and often targeted for POU water treatment interventions [8,9]. However, the results of this study suggest that combined coagulation-flocculation-disinfection POU treatment may select for *Mycobacterium* in treated water, and that POU treatment has the potential to leave viable Mycobacteria in treated, stored household drinking water. These bacteria may form biofilms, creating a continuing source of these bacteria in stored water. 

## 4. Conclusions

A POU coagulant-flocculant-disinfectant treatment that effectively reduced *A. hydrophila* from natural surface waters by 4–7 log_10_ was not effective in reducing Mycobacteria. In both ODF buffer and natural surface water, coagulant-flocculant-disinfectant achieved ~7 log_10_ reduction of *A. hydrophila*, exceeding the POU water treatment bacterial reduction levels of both EPA guidelines (>6 log_10_) and WHO targets (4 log_10_ reduction)In natural waters contaminated with human fecal waste, reductions were lower but still reached 4 log_10_ to meet the WHO performance target. Therefore, POU treatment and safe storage interventions may need to take into account risks from viable NTM in treated stored water and consider alternative treatment processes, such as filtration for physical removal a more effective technology to achieve NTM reductions from water.
